# The HSP90 Inhibitor, AUY-922, Protects and Repairs Human Lung Microvascular Endothelial Cells from Hydrochloric Acid-Induced Endothelial Barrier Dysfunction

**DOI:** 10.3390/cells10061489

**Published:** 2021-06-13

**Authors:** Ruben M. L. Colunga Biancatelli, Pavel Solopov, Betsy Gregory, John D. Catravas

**Affiliations:** 1 Frank Reidy Research Center for Bioelectrics, Old Dominion University, Norfolk, VA 23508, USA; psolopov@odu.edu (P.S.); bgregory@odu.edu (B.G.); jcatrava@odu.edu (J.D.C.); 2 School of Medical Diagnostic & Translational Sciences, College of Health Sciences, Old Dominion University, Norfolk, VA 23508, USA

**Keywords:** acidosis, hydrochloric acid, HCl, endothelial dysfunction, heat shock protein, HSP90 inhibition, AUY-922, acute lung injury

## Abstract

Exposure to hydrochloric acid (HCl) leads acutely to asthma-like symptoms, acute respiratory distress syndrome (ARDS), including compromised alveolo-capillary barrier, and respiratory failure. To better understand the direct effects of HCl on pulmonary endothelial function, we studied the characteristics of HCl-induced endothelial barrier dysfunction in primary cultures of human lung microvascular endothelial cells (HLMVEC), defined the involved molecular pathways, and tested the potentially beneficial effects of Heat Shock Protein 90 (HSP90) inhibitors. HCl impaired barrier function in a time- and concentration-dependent manner and was associated with activation of Protein Kinase B (AKT), Ras homolog family member A (RhoA) and myosin light chain 2 (MLC2), as well as loss of plasmalemmal VE-cadherin, rearrangement of cortical actin, and appearance of inter-endothelial gaps. Pre-treatment or post-treatment of HLMVEC with AUY-922, a third-generation HSP90 inhibitor, prevented and restored HCl-induced endothelial barrier dysfunction. AUY-922 increased the expression of HSP70 and inhibited the activation (phosphorylation) of extracellular-signal regulated kinase (ERK) and AKT. AUY-922 also prevented the HCl-induced activation of RhoA and MLC2 and the internalization of plasmalemmal VE-cadherin. We conclude that, by increasing the expression of cytoprotective proteins, interfering with actomyosin contractility, and enhancing the expression of junction proteins, inhibition of HSP90 may represent a useful approach for the management of HCl-induced endothelial dysfunction and acute lung injury.

## 1. Introduction

Exposure to hydrochloric gas represents a threat to public health. Every year, ~2.5 million metric tons of hydrochloric gas are produced worldwide and employed widely in oil and gas drilling facilities, science laboratories, swimming pools, and illegal drug manufacturing. Hydrochloric acid inhalation provokes a wide range of respiratory symptoms which are directly related to its concentration and duration of exposure [1]. Patients develop cough, shortness of breath, pulmonary edema, and pneumonitis, which can culminate in acute distress respiratory syndrome (ARDS), obstructive respiratory failure and, at higher doses, even death [2]. Usually, respiratory symptoms last for 3–7 days, but several cases of persistent asthma-like conditions, pleural thickening, and pulmonary fibrosis have been described, which can occur even after a single exposure to this dangerous chemical [3,4,5]. This has been replicated in a murine model of hydrochloric acid-instilled mice, which after 30 days culminated in loss of lung alveolar architecture, collagen deposition and pulmonary fibrosis [6,7]. Due to its high solubility, hydrochloric gas dissolves in saliva and mucus of the airways, creating hydrochloric acid (HCl). HCl is a strong acid, capable of easily donating H^+^ ions, provoking direct chemical burns to cells and tissue, causing tissue destruction and alveolar hemorrhage [8], and eliciting a strong inflammatory response. As we have previously shown, HCl increases the number of leucocytes (WBC) in the bronchoalveolar lavage fluid (BALF), mostly monocytes and lymphocytes, and stimulates high levels of interleukins (IL-2, IL-10, IL-6, MCP-1, and TGF-β) [6]. BALF is further characterized by increased proteinosis, reflecting acute damage to the alveolar–endothelial barrier. The increased permeability of endothelial cells, which allows the passage of inflammatory cells and proteins, is believed to be the initial step in the inflammatory response of the lung [9]. There are no antidotes against HCl-induced lung injury as yet, but certain candidates have emerged. Among these, the inhibition of heat shock protein 90 (HSP90), a highly expressed and conserved *chaperone*, has been proposed as a potential therapeutic target in models of lipopolysaccharide (LPS)-, TGF-β-, thrombin-, or VEGF- induced endothelial barrier dysfunction [10,11]. Under physiologic conditions, HSP90 is responsible for the correct folding of more than 400 “client” proteins [12]. Many of these client proteins represent key steps in the pathogenesis of several diseases, so that inhibition of HSP90 represents an exciting area of research in the management of lung injury [13,14], cancer [15], and neurodegenerative disorders [16,17]. In addition, HSP90 exerts a crucial role in the development of inflammation, and HSP90 inhibitors have been shown to modulate the inflammatory cascade and the transcription of acute phase proteins [18]. Therefore, we hypothesized that inhibition of HSP90, by AUY-922 a third generation HSP90 inhibitor, employed either as pre-treatment or post-treatment, could represent an important therapeutic intervention against HCl-induced endothelial barrier dysfunction.

## 2. Materials and Methods

### 2.1. Reagents

Hydrochloric acid (ACS grade), red protein G affinity beads, RIPA buffer, and protease inhibitor cocktail were purchased from Sigma-Aldrich Corporation (St. Louis, MO, USA). AUY-922 was obtained from Selleck Chemicals (Houston, TX, USA). The BCA Protein assay kit was from Pierce Co. (Rockford, IL, USA), and Western blot membranes from GE Healthcare (Chicago, IL, USA). All antibodies used in Western blots and immunoprecipitation have published immunospecificity data available online. Rabbit anti-AKT (9272S), anti-RhoA (67B9), anti-myosin light chain 2 (3672), anti-phospho-MLC2 (3674S), and anti-HSP70 antibodies were purchased from Cell Signaling Technology (Danvers, MA, USA). Mouse monoclonal anti-β-actin was purchased from Sigma-Aldrich Corporation, and secondary IRDye 800CW goat anti-rabbit (926-32211) and IRDye 680RD goat anti-mouse (926-68070) from LI-COR Biosciences (Lincoln, NE, USA). For antibodies used in immunoprecipitation, mouse anti-phosphotyrosine was from Invitrogen, and mouse anti-HSP90 from BD Transduction Laboratories (Franklin Lakes, NJ, USA). For SDS-PAGE: Protogel (30% acrylamide mix) and TEMED were from National Diagnostics (Atlanta, GA, USA), Tris-HCl buffer from Teknova (Hollister, CA, USA), 10% SDS, Trypan Blue 0.4% solution, and ammonium persulfate from Thermo Fisher Scientific (Waltham, MA, USA), and protein dual color standards and tricine sample buffer from Bio-Rad Laboratories.

### 2.2. Cell Culture and Cell Viability

In-house harvested human lung microvascular endothelial cells (HLMVEC) were maintained in M199 media supplemented with 20% FBS and antibiotics/antimycotics as described previously [19]. To assess possible changes in viability, HLMVEC were incubated with different concentrations of AUY-922 (1, 2 or 10 µM). After 24 h, cells were resuspended, Trypan Blue-treated, and counted by hemocytometer. Experiments were run in triplicates, and data are reported as percent of control (vehicle).

### 2.3. Endothelial Barrier Function

HLMVEC were seeded on electrode arrays (8W10E+), and endothelial barrier integrity was estimated by the electric substrate impedance sensing (ECIS) technique, using an ECIS model 1600R ζθ instrument (Applied BioPhysics). Experiments were conducted when a stable resistance was maintained above 800 Ω, as we have previously published [20]. Experiments were performed in triplicates and repeated at least three times. Resistance values were collected and normalized to each well’s value at t = 0. Data are presented as mean values (±SEM). Results were considered significant when *p* < 0.05 with one-way ANOVA and Tukey’s post-test.

### 2.4. Protein Isolation and Western Blots

HLMVEC were cultured in 100 mm dishes until 90–95% confluency. For pre-treatment, cells were incubated with either 2 µM AUY-922 or vehicle (saline) and after 24 h were exposed to HCl (0.01 N) for 1 h before protein isolation. For post-treatment, confluent dishes were instilled with HCl (0.01 N), and after 5 min treated with either AUY-922 or vehicle (saline) for 3.5 h. To stop the experiment, dishes were placed on ice and washed 3× with ice-cold PBS. PBS was removed, and ice-cold lysis buffer was added (RIPA with protease inhibitor cocktail 1:100). Cells were scraped and the cell suspension was transferred to a microcentrifuge tube. Tubes were placed at 4 °C for 30 min under continuous agitation. Protein concentration was estimated by the BCA protein assay. Equal volumes of tricine buffer with 2% 2-mercaptoethanol were added to samples containing equal amounts of protein. Proteins were denaturated by 10 min boiling at 100 °C. Protein lysates were then subjected to SDS-PAGE in 8%, 10%, and 12% sodium dodecyl sulfate Tris-HCl gels. Proteins were transferred onto nitrocellulose membranes and blocked for 1 h at room temperature in 5% non-fat dry milk in tris-buffered saline containing 0.1% Tween 20. Membranes were incubated overnight at 4 °C with specific antibodies, washed 4×, and incubated for 1 h at room temperature with appropriate peroxidase-conjugated secondary antibody. Bands were detected by digital fluorescence imaging (LI-COR Odyssey CLx). Densitometric evaluation of the bands was made using ImageJ software (National Institutes of Health, Bethesda, MD, USA). Β-actin was used as a loading control.

### 2.5. Immunocytochemistry

Round coverslips (Thermo Fisher Scientific) were placed in 12-well plates, soaked in 70% ethanol for 15 min, and dried under the hood. The cover glasses inside the wells were covered with 1 mL 0.2% gelatin and incubated 30 min at 37 °C. Excess gelatin was aspirated, and 500 µL of cell suspension containing 6 × 10^5^ HLMVEC was placed on top. Coverslips with at least 90% confluent HLMVEC were fixed in 4% paraformaldehyde in PBS for 10 min, washed 3×, permeabilized with 0.1% Triton-X 100 in TBS for 10 min, triple washed in PBS, and blocked with 5% BSA in 0.1% TWEEN overnight at 4 °C. The next day, coverslips were moved to a wet camera and incubated with VE-cadherin antibody (Abcam, 1:50 dilution) in blocking buffer at 4 °C for 24 h. After 5× washes in PBS, cells were incubated with Alexa Fluor 488 goat anti-rabbit secondary antibody (Thermo Fisher Scientific, dilution 1:500) in the dark at RT for 1 h and then washed 5×. F-Actin was stained using Texas Red—X phalloidin (Life Technologies, diluted 1:300). At the counterstaining stage, cells were incubated with 300 µM DAPI for 5 min in the dark. One drop of mounting media (ProLong Plus, Thermo Fisher Scientific) was added onto pre-cleaned microscope slides (Superfrost Plus, Thermo Fisher Scientific), placed over the top of the coverslips facing down, extra PBS was removed from the edges, and final slides were placed in the dark at RT for overnight drying. Confocal analysis was performed with an Olympics Fluoview FV10i microscope, and pictures were analyzed with ImageJ.

### 2.6. Statistical Analysis

Statistical significance of differences among groups was determined by the one-way analysis of variance (ANOVA) followed by the Bonferroni or Tukey post hoc tests using GraphPad Prism Software (GraphPad Software, San Diego, CA, USA). Differences among groups were considered significant at *p* < 0.05.

## 3. Results

### 3.1. HCl Elicits a Concentration-Dependent Decrease in TER

**Figure 1:**
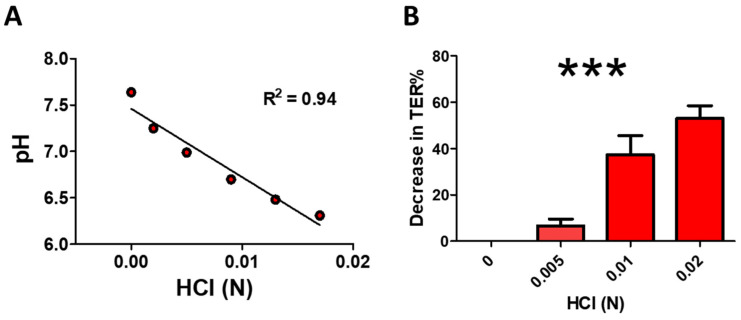
Hydrochloric acid (HCl) elicits a concentration-dependent decrease in pH (**A**) and trans-endothelial resistance (TER); (**B**)). Line in (**A**) represents linear regression analysis (***: *p* < 0.001; Pearson R^2^ coefficient 0.94). The maximal decrease in TER in (**B**) was plotted as a percentage of the basal resistance in the absence of HCl (*n* = 3, means ± SEM; ***: *p* < 0.001, one-way ANOVA with Tuckey’s).

Increasing HCl concentrations linearly decreased pH in Dulbecco Modified Eagle Medium (DMEM pH 7.64), the medium used in human lung microvascular endothelial cells (HLMVEC) (Figure 1A). Furthermore, increasing concentrations of HCl decreased trans-endothelial resistance (TER) of HLMVEC monolayers in a concentration-dependent manner (Figure 1B).

### 3.2. Pre-Treatment with the HSP90 Inhibitor, AUY-922, Protects HLMVEC from HCl-Induced Endothelial Barrier Dysfunction

**Figure 2:**
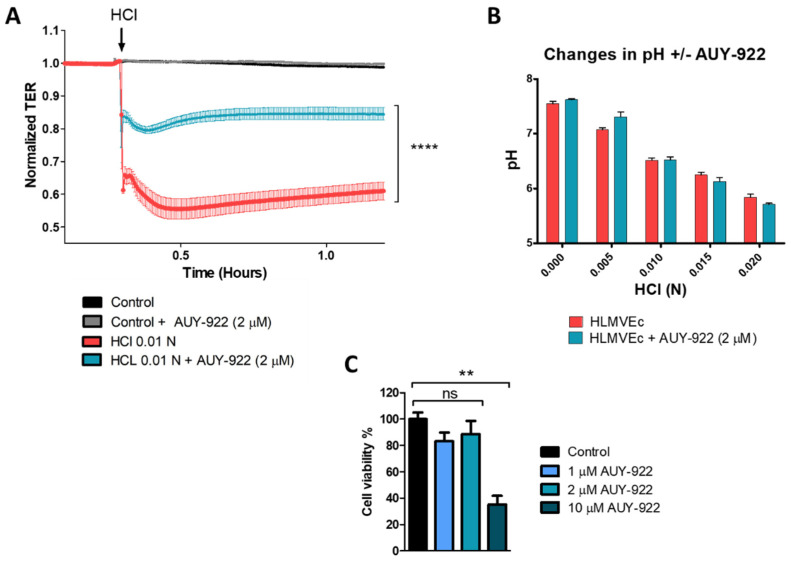
Pre-treatment with the HSP90 inhibitor, AUY-922, prevents HCl-mediated hyperpermeability of HLMVEC monolayers. HLMVEC were seeded on 8W10E+ arrays until confluent, i.e., until stable resistance was achieved (>800 Ω). (**A**) HLMVEC were treated with AUY-922 (2 µM) for 24 h and then challenged with 0.01 N HCl (arrow). Resistance was recorded continuously at 10 s intervals and normalized to time = 0 h. (**B**) The HSP90 inhibitor, AUY-922, added to the media in a final concentration of 2 µM, did not affect HCl-induced decrease in pH. (**C**) Cell viability of HLMVEC incubated for 24 h with different concentrations of AUY-922 (1, 2, and 10 µM). Means ± SEM; *n* = 3. **: *p* < 0.01; ****: *p* < 0.0001 by one-way ANOVA with Bonferroni’s post-test.

HLMVEC were seeded on electrode arrays (8W10E+) till confluent. The HSP90 inhibitor, AUY-922, was added to a final concentration of 2 μM, 24 h prior to the addition of HCl (0.01 N). The HCl dose was chosen from results shown in Figure 1B, as it reduces TER by approximately 40%. HCl produced an immediate, time-dependent decrease in TER, which was significantly reduced in cells pre-treated with AUY-922 (Figure 2A). To make sure that the protective effect of AU-922 was not due to buffering and restoration of normal pH, we measured pH values over a range of HCl concentrations, in the presence and absence of 2 μM AUY-922. There was no effect of the HSP90 inhibitor on pH (Figure 2B). Furthermore, 24 h incubation with 1 or 2 µM AUY-922 did not affect cell viability (Figure 2C).

### 3.3. Pre-Treatment with AUY-922 Induces Cytoprotection

**Figure 3:**
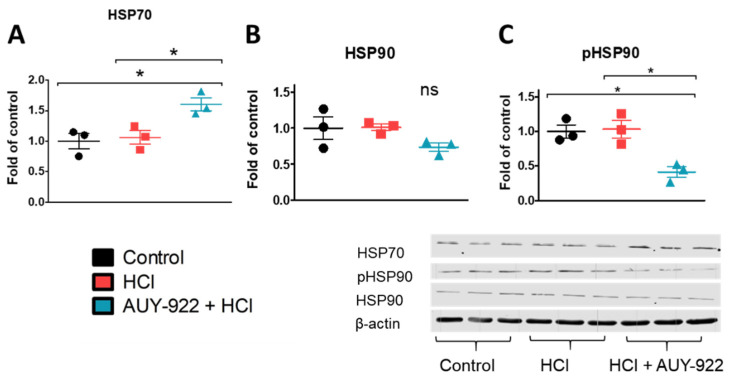
The HSP90 inhibitor, AUY-922, promotes the overexpression of cytoprotective mechanisms involved in endothelial barrier integrity. (**A**) HSP70 expression increased, (B) HSP90 expression did not change, while (**C**) p-HSP90 (active HSP90) decreased after 24 h incubation with AUY-922. HLMVEC grown in 100 mm culture dishes were pre-treated for 24 h with 2 µM AUY-922 and then exposed to 0.01 N HCl. One hour later, cells were lysed, and samples were prepared for protein analysis. Means ± SEM; *n* = 3; *: *p* <0.05, with one-way ANOVA and Tukey’s.

To explore mechanisms potentially responsible for the barrier protective effects of HSP90 inhibition, we analyzed pathways involved with cell survival, cytoskeletal rearrangements, and tight junction expression. The inhibition of HSP90 for 24 h resulted in the overexpression of HSP70, a well-known cytoprotective molecule. This effect was accompanied by the simultaneous inhibition of the phosphorylated (activated) form of HSP90 (p-HSP90), while no changes were observed in the levels of total HSP90 (Figure 3A–C).

### 3.4. Pre-Treatment with AUY-922 Prevents the HCl-Induced AKT Phosphorylation

**Figure 4:**
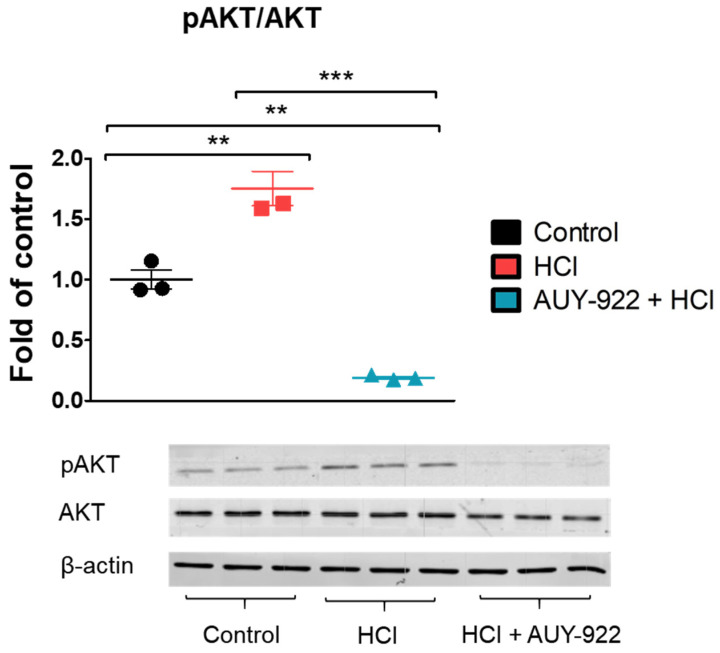
The HSP90 inhibitor, AUY-922, prevents the HCl-induced AKT phosphorylation. HLMVEC grown in 100 mm cultured dishes, were pre-treated for 24 h with 2 µM AUY-922 and then exposed to 0.01 N HCl. One hour later, cells were lysed, and samples were prepared for protein analysis by Western Blotting. Means ± SEM; *n* = 3; **: *p* <0.01; ***: *p* < 0.001 with one-way ANOVA and Tukey’s.

Protein kinase B (AKT) is critically involved in the integrity of the endothelial barrier. Pre-treatment with 2 µM AUY-922 for 24 h completely inhibited AKT phosphorylation, which was increased by HCl (Figure 4).

### 3.5. Pre-Treatment with AUY-922 Modulates the Activation of Cytoskeletal Proteins and the Expression of Tight Junction Proteins

**Figure 5:**
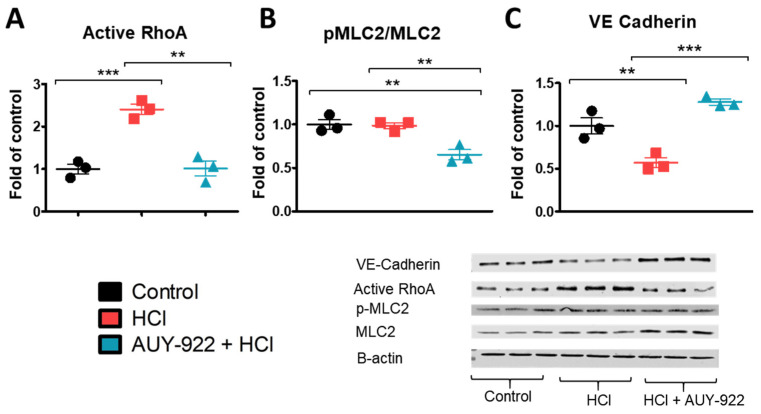
The HSP90 inhibitor, AUY-922, modulates HCl-induced cytoskeletal rearrangements and promotes the expression of adherent junction proteins in HLMVEC. (**A**) The activation of RhoA induced by HCl was completely inhibited after 24 h incubation of HLMVEC with AUY-922 (2 µM). (**B**, **C**) AUY-922 inhibited MLC2 activation and increased the expression of VE-cadherin. HLMVEC grown in 100 mm cultured dishes were pre-treated for 24 h with vehicle or 2 µM AUY-922 and then exposed to 0.01 N HCl. One hour later, cells were lysed, and samples were prepared for protein analysis by Western blotting. Means ± SEM; *n* = 3; **: *p* < 0.01; ***: *p* < 0.001 with one-way ANOVA and Tukey’s.

The HCl-induced endothelial barrier dysfunction may be consequent to rearrangements in the expression of cytoskeletal proteins and tight junction proteins. Pre-treatment with the HSP90 inhibitor AUY-922 for 24 h prevented the HCl-induced activation of Ras homolog family member A (RhoA), reduced the phosphorylation of myosin light chain 2 (MLC2), and increased the expression of VE-cadherin (Figure 5A–C).

### 3.6. Pre-Treatment of HLMVEC with AUY-922 for 24 h Prevents the HCl-Induced Loss of Plasmalemmal VE-Cadherin

**Figure 6:**
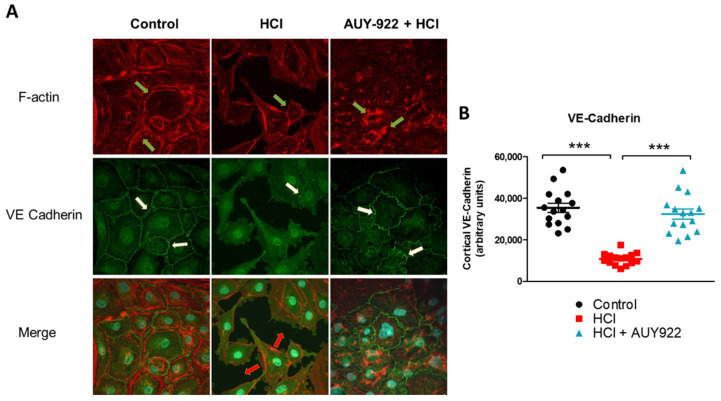
AUY-922 protects HLMVEC against HCl-induced VE-cadherin and F-actin reorganization. (**A**) HLMVEC were pre-treated with either vehicle (PBS) or AUY-922 (2 µM) for 24 h and then exposed to 0.01 N HCl for 1 h. Cells were fixed and stained for VE-cadherin, F-actin, and DAPI. (**B**) Quantification of cortical VE-cadherin staining. Means ± SEM; *n* = 3; ***: *p* < 0.001 with one-way ANOVA and Tukey’s.

HLMVEC were seeded onto sterile, 0.2% gel-coated glass coverslips. When confluent, they were pre-treated for 24 h with either PBS (control) or 2 µM AUY-922, before instillation of 0.01 N HCl or vehicle. One hour later, the cells were fixed, permeabilized, blocked, and incubated with VE-cadherin or F-actin antibody and counterstaining antibodies. HCl created gaps among confluent cells, altered the cellular shape, and reduced the cortical expression of VE-cadherin and F-actin. Pre-treatment with AUY-922 protected HLMVEC from HCl-mediated damage, preserving VE-cadherin expression and concentrating F-actin in the perinuclear areas (Figure 6A). Quantification of cortical VE-cadherin staining further demonstrated that AUY-922 prevented HCl-induced loss of VE-cadherin staining (Figure 6B).

### 3.7. Post-Treatment with AUY-922 Restores HCl-Induced Hyperpermeability

**Figure 7:**
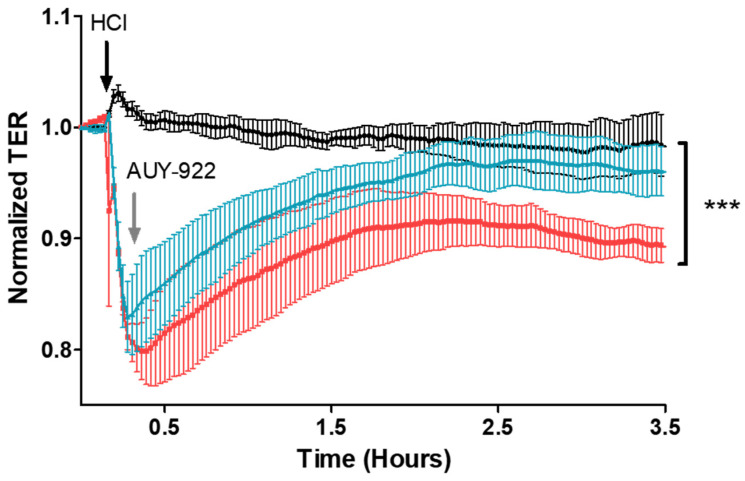
Post-treatment with the HSP90 inhibitor, AUY-922, repairs HCl-induced HLMVEC barrier dysfunction. HLMVEC were seeded on 8W10E+ arrays; when confluent (R > 800 Ω), 0.01 N HCl (final concentration) was added (black arrow). At the nadir of the observed TER values, 2 µM (final concentration) AUY-922 (grey arrow) or vehicle was added. TER was recorded continuously with an interval time of 10 s and normalized to time = 0 h. Post-treatment with AUY-922 completely restored endothelial barrier function. Means ± SEM; *n* = 3. ***: *p* < 0.001 between HCl and HCl + AUY groups, one-way ANOVA with Bonferroni’s post-test.

We then investigated if inhibition of HSP90 would repair HCl-induced HLMVEC barrier dysfunction. Cells were first exposed to 0.01 N HCl and, when they reached the nadir of trans-endothelial resistance, saline or AUY-922 was added to the wells, at a final concentration of 2 µM. Cells treated with the HSP90 inhibitor, AUY-922, displayed complete recovery in trans-endothelial resistance compared to cells instilled with HCL and treated with vehicle (Figure 7).

### 3.8. Post-Treatment with AUY-922 Inhibited the Activation of AKT and ERK

**Figure 8:**
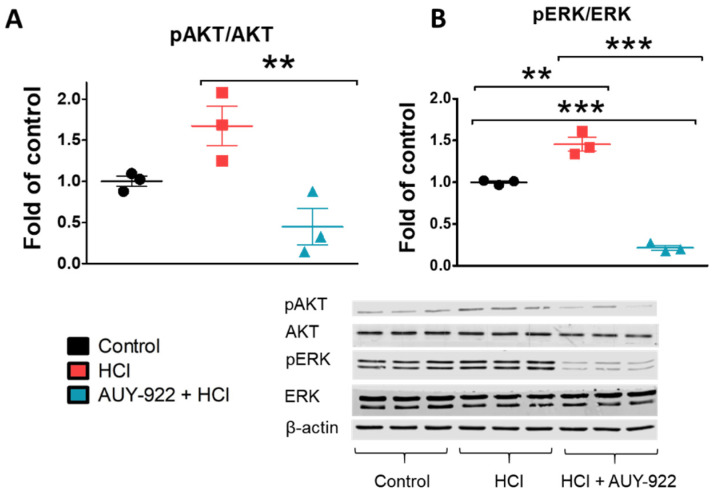
Post-treatment with the HSP90 inhibitor, AUY-922, abolishes the phosphorylation of crucial kinases involved in endothelial integrity. (**A**,**B**) pERK/ERK and pAKT/AKT ratios in cell lysates were analyzed by Western blotting. 100 mm culture dishes of confluent HLMVEC received 0.01 N HCl and after 5 min were treated with either 2 µM AUY-922 or vehicle. Three hours later, cells were lysed and proteins were analyzed. Means ± SEM; *n* = 3; **: *p* < 0.01; ***: *p* < 0.001 with one-way ANOVA and Tukey’s.

HLMVEC were seeded in 100 mm dishes until confluent. Five minutes after addition of 0.01 N HCl, AUY-922 (2 μM) was added; three hours later, dishes were put on ice, cells lysed, and proteins analyzed. HCl activated (phosphorylated) both ERK and AKT; post-treatment with AUY-922 completely inhibited both ERK and AKT phosphorylation (Figure 8A, B).

## 4. Discussion

Exposure to HCl provokes acute lung injury, which, depending on duration of exposure and HCl concentration, could prove lethal. HCl acute toxicity is mediated by a direct chemical burn, followed by leucocyte infiltration and activation of various proinflammatory cytokines [6]. Alveolar hemorrhage, increased lung proteinosis, and cell infiltrates into the BALF are common features as a result of damage to the endothelial–alveolar barrier [10]. If the endothelial injury persists, fluids move to the alveolar surface and impede oxygen exchange, provoking acute respiratory distress syndrome (ARDS). Thus, targeting endothelial cell dysfunction is an intriguing approach to acute lung injury. We investigated the characteristics of HCl-mediated endothelial injury through continuous, real-time analysis of changes in trans-endothelial electrical resistance (TER). TER reflects the level of endothelial cell integrity and thus the function of the endothelial barrier. When toxins, chemicals, or drugs damage the endothelial barrier, gaps occur between cells and the passage of proteins, and liquid is facilitated. Exposure of HLMVEC to HCl led to a fast, concentration-dependent perturbation of endothelial barrier function (Figure 1 and Figure 2). As a strong acid, HCl is capable of reducing the pH of the media and of provoking cell injury. A pH reduction to 6.7 was enough to provoke changes in TER of 40%. However, inhibition of HSP90, obtained by incubating cells with AUY-922 before HCl instillation (pre-treatment), resulted in decreased damage to endothelial cells and preserved the function of the monolayer, without affecting pH (Figure 2). These protective effects were also visible when AUY-922 was employed after HCl exposure (post-treatment) and achieved complete recovery of endothelial barrier function (Figure 7). To study the therapeutic effects of AUY-922 on the endothelium, we analyzed two well-known HSP90 client proteins that would be affected by drugs that act on HSP90’s chaperone function. Protein kinase B (AKT) and extracellular-signal regulated kinase (ERK) were activated by HCl-induced injury, but this activation was completely blocked by AUY-922 when employed either as pre-treatment or post-treatment (Figure 4 and Figure 8). Both kinases are involved in endothelial cell function. AKT modulates apoptotic pathways and its signaling regulates angiogenesis via the PI3/AKT/mTOR pathway [21]. AKT phosphorylation, in addition, regulates nitric oxide (NO) synthase (eNOS) activity and cell proliferation and has been related to endothelial dysfunction observed in models of hypertension [22]. ERK is involved in multiple signaling pathways and has been shown to regulate vascular proliferation in response to insults [23].

TER data suggest that distinct cellular rearrangements occur when the monolayer is exposed to HCl, alone or in the presence of AUY-922. These changes were reflected in the activation of RhoA by HCl and the therapeutic modulation of RhoA and pMLC2 by the HSP90 inhibitor (Figure 5). AUY-922 was also able to promote increased expression of VE-cadherin (Figure 5) and prevent F-actin reorganization induced by HCl (Figure 6). HSP90 inhibitors preferentially target the activated—Tyr phosphorylated—form of HSP90 and also induce overexpression of HSP70 (Figure 3). This is part of the heat shock factor-1 (HSF-1) mediated response produced by HSP90 inhibition, and it is considered a cytoprotective mechanism [24].

**Figure 9:**
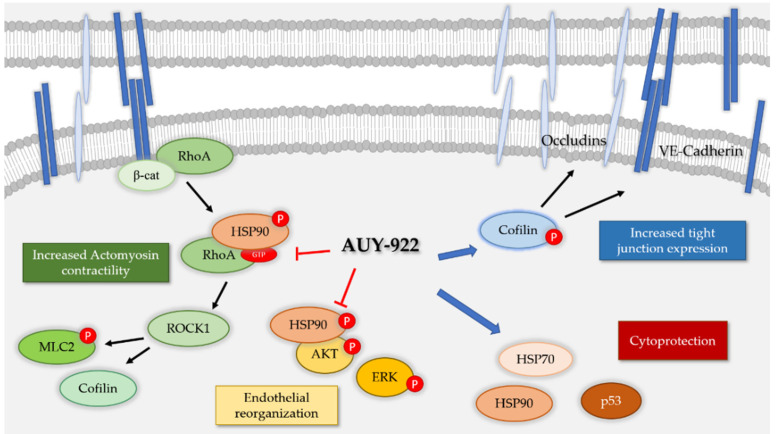
Proposed mechanisms of action of the HSP90 inhibitor AUY-922 in the endothelium. Ras homolog family member A (RhoA) actively maintains cadherin expression but when activated is responsible of increased actomyosin contractility via cofilin and myosin light chain 2 (MLC2). Protein kinase B (AKT) and extracellular signal regulated kinase (ERK) are similarly involved in endothelial reorganization and injury. AUY-922, by inhibiting the phosphorylated form of HSP90, block actomyosin contractility and endothelial rearrangement. In addition, AUY-922 favors the expression of cytoprotective p53 and HSP70. Finally, increasing the phosphorylated form of cofilin promotes the expression of tight junction proteins, such as VE-cadherin and occludin.

The inhibition of HSP90 by other HSP90 inhibitors, 17-AAG and 17-DMAG, has been shown to be beneficial in an in vitro model of LPS-induced endothelial injury [10]. HSP90 inhibitors modulate endothelial inflammation via multiple mechanisms, including the inhibition of the IKBα promoter [25] as well as their actions on Sirtuin 2 [26]. These anti-inflammatory effects, together with the modulation of RhoA activity, MLC2 phosphorylation, and the increased expression of tight junction proteins represent important features of HSP90 inhibition that are crucial as endothelial barrier protectants (Figure 9).

While we have already shown that HSP90 inhibition could represent a new target for antifibrotic therapy [13,27] in HCl-induced chronic lung injury, this is the first report that suggests HSP90 inhibition may be a promising approach for the treatment of HCl acute endothelial injury. In fact, there are no FDA-approved drugs for the treatment of HCl-toxicity. This study, however, has limitations. The ECIS assay represents a useful but simplified model of capillary permeability. The cells are seeded on electrodes and lack all the cellular complexity that defines microvascular anatomy. Still, this model could be of use for future research regarding pathological conditions with persistent acidosis, usually observed in the critically ill (e.g., diabetic ketoacidosis, sepsis, respiratory failure) and characterized by high morbidity and mortality.

## 5. Conclusions

Hydrochloric acid (HCl) is a toxic chemical whose exposure is related to severe, potentially lethal, acute and chronic toxicity. Here we show that HCl provokes human lung microvascular endothelial cell barrier dysfunction via the activation of ERK and AKT and the consequent activation of cytoskeletal proteins. AUY-922, a third-generation HSP90 inhibitor, prevented and restored HCl-induced endothelial barrier hyperpermeability by modulating ERK and AKT phosphorylation, preventing increases in RhoA, and promoting the expression of VE-Cadherin. The inhibition of HSP90 represents a promising new therapeutical target for HCl-induced acute lung injury and endothelial dysfunction.

## Data Availability

Derived data supporting the findings of this study are available from the corresponding author on request.
